# Inferring space from time: On the relationship between demography and environmental suitability in the desert plant *O*. *rastrera*

**DOI:** 10.1371/journal.pone.0201543

**Published:** 2018-08-09

**Authors:** Carolina Ureta, Carlos Martorell, Ángela P. Cuervo-Robayo, María C. Mandujano, Enrique Martínez-Meyer

**Affiliations:** 1 Departamento de Ecología y Recursos Naturales, Facultad de Ciencias, Universidad Nacional Autónoma de México, Mexico City, Mexico; 2 Departamento de Zoología, Instituto de Biología, Universidad Nacional Autónoma de México, Mexico City, Mexico; 3 Comisión Nacional para el Conocimiento y Uso de la Biodiversidad (CONABIO), Insurgentes Sur-Periférico, Tlalpan Mexico City, Mexico; 4 Departamento de Ecología Evolutiva, Instituto de Ecología, Universidad Nacional Autónoma de México, Mexico City, Mexico; Universita degli Studi di Napoli Federico II, ITALY

## Abstract

Demographic analyses and ecological niche modeling (ENM) are two popular tools that address species persistence in relation to environmental conditions. Classic demography provides detailed information about the mechanisms that allow a population to grow or remain stable at a local scale, while ENM infers distributions from conditions suitable for species persistence at geographic scales by relating species’ occurrences with environmental variables. By integrating these two tools, we may better understand population processes that determine species persistence at a geographic scale. To test this idea, we developed a model that relates climate to demography of the cactus *Opuntia rastrera* using 15 years of data from one locality. Using this model we determined the geographic area where populations would have positive growth rates given its climatic conditions. The climate-dependent demographic model showed poor performance as a distribution model, but it was helpful in defining some mechanisms that determine species’ distributions. For instance, high rainfall had a negative impact on the population growth rate by increasing mortality. Rainy areas to the west of the distribution of *O*. *rastrera* were identified as unsuitable both by our climate-dependent demographic model and by a popular ENM algorithm (MaxEnt), suggesting that distribution is constrained by excessive rains due to high mortality. Areas projected to be climatically suitable by MaxEnt were not related with higher population growth rates. Instead, we found a strong correlation between environmental distance to the niche centroid (center of the niche hypervolume, where optimal conditions may occur) and population growth rate, meaning that the niche centroid approach is helpful in finding high-fitness areas.

## Introduction

Demographic analyses and ecological niche modeling (ENM) are two ecological tools that address species persistence in different ways. Demographic studies investigate how demographic processes (e.g., survival, growth and reproduction) affect population growth rate (*λ*) to provide detailed information about the mechanisms that allow a population to grow or remain stable given the conditions that occur at a local scale [1]. ENM identifies environmental conditions related with species persistence and provides a measure of the likelihood that a species will persist on a larger scale (species’ geographic ranges). While demographic analyses are limited by scale, ENM does not attempt to explain the biological mechanisms that determine species performance. Thus the value of integrating these two tools has previously been identified whereby studies have integrated population data with spatial analyses to understand species’ range-wide population processes, such as performance or spatially explicit extinction risk, resulting in more effective conservation strategies [2,3,4,5,6]. Combining a detailed demographic analysis with ENMs is expected to increase our understanding of species’ biogeography and give more informative results for species conservation.

Following Hutchinson [7], the ecological niche of a species is the hypervolume of environmental conditions where the species can persist through time. Conditions close to the boundaries of the hypervolume are expected to be less favorable for species’ persistence, whereas conditions more central in the hypervolume are expected to be closer to the species optimum [8]. For instance, it has been found that population density diminishes with distance from the niche centroid [4]. In demographic terms, niche can be envisaged as the environmental conditions where *λ* ≥ 1, (i.e., the conditions where population does not decline to extinction), and in ENM, niche can be considered as the set of environmental conditions that are suitable enough to permit species occurrence. Thus, we may expect suitablity and *λ* to be greater toward the niche centroid.

Analysis of demographic processes has previously been integrated into ENM to gain insight on extinction risk under climate change [2,4,9]. Potential reductions in distribution do not translate directly into extinction probability and thus demographic population viability models are required [2]. In 2015, a method was proposed to create distribution models based on short-term demographic studies of *Protea repens* throughout its range [9]. In this method, demographic processes of the populations were related to climatic variables to extrapolate population performance (population growth rate) across the species’ distribution.

We propose an alternative strategy: instead of having several localities studied over one-year [5], we created a distribution model from one locality based on a 15-year-long demographic study. This procedure makes use of the climatic variability observed over time instead of climate differences between sites. The basic idea is to relate demographic processes with climatic conditions observed over 15 years and integrate this data into a climate-dependent demographic model. We can then use this model to estimate the *λ* values under different conditions and to determine the geographic range in which the species can persist given the climate of the range. We expect to capture enough climatic variability over a 15-year period to project the dynamics of population onto species’ distribution areas. Desert systems are particularly appropriate for this purpose because they experience a large interannual climatic variability [10]. *Opuntia rastrera* is characteristically found in desert environments and climate has previously been shown to play an important role in its population dynamics [11], making our 15-year dataset ideal for testing our new strategy.

Here, we construct a climate-dependent demographic model and an ENM to investigate the benefits of integrating the two approaches to gain insights into the mechanisms that determine species distribution. This involved the following four steps: a) analyze the potential of climate-dependent demographic models to predict species’ distribution areas based on the climatic characteristics of the landscape; b) assess the demographic processes that determine species’ distributions; c) test for a relationship between suitability values estimated with a popular ENM algorithm (MaxEnt) and values of *λ* obtained from the climate-dependent demographic model; and d) test the concept that areas presenting higher *λ* are closer to the niche centroid.

## Materials and methods

### Species under study and fieldwork

The fieldwork took place in the natural protected area of Mapimí, Durango, in northern Mexico (26°41'08 N, 103°44' W, altitude of 1160 m a.s.l.). This study was carried out in strict accordance with SEMARNAT, the governmental authority for natural protected areas in Mexico. No protected species were sampled. Data from the local weather station between 1975 and 2005 indicates a semi-arid climate with an annual precipitation of 465 **±** 50 mm (mean **±** s.d.) and a mean annual temperature of 17 **±** 4.5°C.

The study species is *O*. *rastrera* F.A.C. Weber (Cactaceae), which grows as a prostrate shrub. Cladodes (racquet like stems) are oval shaped and create long chains. It has sexual and clonal reproduction, but in this study we only considered clonal reproduction. Clonality was observed almost every year and seedlings were rarely observed, died shortly after germination, and consequently make a negligible contribution to population dynamics in our data set [12]. Individuals clone by dropping one or more cladodes on the ground, which may produce roots and become established. The population dynamics of the species was evaluated from 1991 to 2007 (15 annual transitions) spanning considerable climatic variation (see Table A in S1 File).

Four plots of 20 × 20 m were randomly established in a 4-ha area where *O*. *rastrera* dominates. The population was surveyed yearly in early spring. The number of individuals studied per year varied from 553 to 1138, while the number of clones identified varied from 30 to 72 as indicated in S1 File. We established four plots because we expected that sampling more than one area would include plants with different genotypes and thus more thoroughly represent species diversity. Given the great complexity involved in estimating the growth functions, we did not include plots when fitting kernel functions (i.e., when all individuals are assumed to be independent). This assumption would potentially affect *P* values, which is not a problem as no hypotheses were tested when fitting vital rates.

Plants were tagged and the following information was recorded for each: number of cladodes, flowers and fruits produced, seedling establishment, vegetative propagation by cladode rooting and plant mortality. Seedlings produced sexually were readily distinguishable from those produced clonally [12]. Cladodes were marked individually such that in the case that one of them fell, it would be possible to identify which plant it came from.

### Modeling distributions

We projected the *O*. *rastrera* ENM to a geographic area using existing records and terrestrial ecorregions instead of political boundaries [13,14,15]. This area represents the regions where the species has historically had access climatically and according to its dispersal and colonizing abilities [13]. Within this area we used climate surfaces representing monthly mean values of minimum and maximum temperature and precipitation for the period 1980–2009 (Cuervo-Robayo et al., submitted) (1-km^2^ resolution, WGS84 geographic projection). From these climate surfaces, we derived 19 bioclimatic variables [16] that represent information about annual, seasonal and extreme climatic trends. We used the R platform [17] to carry out the *biovars* function of the *dismo* package [18] and generate the bioclimatic variables. To reduce collinearity, we conducted a Pearson’s correlation (cut-off Pearson’s 0.80 [18,19,20,21] and selected a final set of variables that were biologically relevant to the species: namely Bio1-mean annual temperature, Bio10-mean temperature of the warmest quarter, Bio11-mean temperature of the coldest quarter, Bio12-annual precipitation, Bio13-precipitation of the wettest month, Bio14-precipitation of the driest month, Bio16-precipitation of the wettest quarter and Bio19-precipitation of the coldest quarter.

### Climate-dependent demographic model

Integral projection models (IPMs) have become the standard tool for linking demography and environmental variables. This is because vital rates are described by functions with few parameters, into which it is easy to incorporate environmental data as additional variables. In this particular study, we measured the size of cacti as the number of cladodes in the individual (a discrete variable); this created a transition matrix and not strictly an IPM. However, our model was similar to integral projection models in two respects:

1. Vital rates were modeled as nonlinear functions of the exact size (number of cladodes) of plants and not estimated from the observed fraction of individuals that change size categories as is usual in matrix projection models (MPM). This procedure avoids any loss of information about the individuals’ heterogeneity [22], reduces the number of parameters to be estimated, provides better estimates for poorly represented sizes than classic parameterization of MPMs, and performs well despite small sample sizes [23]. In our case, these considerations are particularly important because we set the interval of possible sizes between one and 100 cladodes, and not enough data was available to reliably estimate each entry of the resulting 100 × 100 matrix.
2. As in IPMs, we summarized survival, growth and fecundity in a complex function–or kernel–from which the elements *k*_*y*,*x*_ of the transition matrix **K** can be estimated. Previous research [23] showed that this approach produces much better estimates of *k*_*y*,*x*_ than traditional estimation procedures based on data from separate categories. The values of *k*_*y*,*x*_, transitions of individuals from size *x* at time *t* to size *y* and at time *t*+1, were calculated as follows:

$$k_{y,x}=s_{x}g_{y,x}+f_{y,x}$$ (1)

where *s*_*x*_ is the survival probability of a plant having *x* cladodes; *g*_*y*,*x*_ is the probability a plant with *x* cladodes has of producing *y* cladodes after one year; and *f*_*y*,*x*_ represents the fecundity as the number of clones with *y* cladodes produced by a mother with a size *x*. These values were calculated from a series of underlying functions: *s*(*x*) and *g*(*x*,*y*) for survival and growth, respectively, plus *M*(*m*|*y*,*x*), *C*(*c*|*m*) and *F*(*l*|*m*,*c*) (see below) for fecundity. Here, we only present the general structure of these functions. For further details on the model and fitting procedures see S1 and S2 Files; the AIC values used for model selection can be found in Tables A-D in S3 File.

Survival probability was modeled as a logistic function between 0 and 1. We used the two functions *b*(*x*), the number of cladodes produced, and *d*(*x*), the number of cladodes dropped, to model the final number of cladodes in an *x*-sized plant. The functions *b*(*x*) and *d*(*x*) represented negative-binomial distributions, which were exponential functions of *x*. Thus, the mean number of cladodes of a plant with size *x* after one year was E(*y|x*) = *x* + E(*b*(*x*))–E(*d*(*x*)), where E is the mathematical expectation. The sum in this formula implies that the distribution of *y* was a convolution of two negative binomial distributions.

For fecundity we used three probability distributions: (1) *M*(*m*|*y*,*x*), the probability that *m* cladodes were dropped by a mother plant with *x* cladodes that had *y* cladodes after one year and had established successfully; this function followed a zero-inflated negative binomial distribution. (2) *C*(*c|m*), the probability that the *m* cladodes are partitioned into *c* clones, which followed a zero-truncated Poisson distribution. (3) *F*(*l*|*m*,*c*), a geometric distribution that described the fraction of the *m* cladodes that end up in the *l*^th^ clone (*l* = 1,2,…,*c*). From this function, we calculated the size of each clone and used this figure to obtain *f*_*y*,*x*_ from the theory on mathematical expectation because *M*(*m*|*y*,*x*) and *C*(*c|m*) are probability distributions (see S1 and S2 Files).

We estimated the kernel parameters for each study year and constructed the respective **K** matrix (see Tables A and B in S4 File for parameter values). This set of annual matrices was incorporated in a stochastic model, from which we obtained the long-term (geometric mean) population growth rate *λ*_*s*_ and the stable size structure. The population growth rate was biologically reasonable (*λ*_*s*_ = 1.005), and the stable population structure resembled closely the observed one, suggesting that our model captured the population dynamics accurately (see Figure A in S1 File and S2 File).

To incorporate the effects of climate on vital rates, we analyzed the relationship between the parameters of the functions *g*(*x*,*y*), *s*(*x*), *M*(*m|y*,*x*) and *C*(*c|m*) for each of the 15 study years with the eight selected bioclimatic variables estimated for the respective year using data from the local meteorological weather station via generalized linear models (S5 File) (Note that *F*(*l|m*,*c*) was estimated using the pooled data for all years due to insufficient data and thus did not change across years). Four bioclimatic variables (mean temperature of the warmest quarter, annual precipitation, precipitation of the wettest month and precipitation of the coldest quarter) had significant relationships with vital rates (S5 File).

Given that it was possible to relate climate with different vital rates, we projected the demographic dynamics over the study region. For this, we obtained the values of the four bioclimatic variables for each 1-km^2^ cell in the study region and used them to calculate the values of the kernel parameters. From this kernel, the matrix **K** was obtained and its dominant eigenvalue was calculated as a measure of the deterministic *λ*, which is also a way to refer to the fitness of the population. With this information, it was possible to produce a potential distribution map created with the species population growth rate of species’ fitness (Stearns 2000), assuming that higher *λ* values are related to higher presence probability.

It is important to note that our estimations of *λ* are based on the assumption of no interannual variability because the climatic layers available are climatic averages (1980–2009). If variability is included in demographic models, it frequently reduces the long-term population growth rate. Thus, it is likely that only populations with a deterministic *λ* greater than one can remain viable in the presence of variability (i.e., their long-term growth rate is greater than one despite the fact that *λ* decreases). Even when we had *λ* values higher than 1.05, we decided to use the true skill statistic (TSS) metric [24] to evaluate the threshold in the distribution model based on demography that minimized the omission and commission errors with several thresholds (*λ* = 1, *λ* = 1.01, *λ* = 1.02, *λ* = 1.03, *λ* = 1.04 and *λ* = 1.05). We evaluated the discrimination performance of predictive models with the “area under the receiving operating characteristic curve” (AUC: [25]. AUC scores closer to 1 represent models that predict presence correctly, and AUC scores closer to 0.5 represent models that do not differ significantly from random.

We also analyzed the effect of different demographic processes on population growth by calculating elasticities of *λ*. As usual, sensitivity was defined as ∂ln*λ*/∂lnθ, where θ is any parameter in the projection model [1,26]. The elasticity of individual parameters in the kernel is relatively difficult to interpret, so here we calculated the elasticity of whole functions *f* in the kernel. To do so, we defined θ to be a constant that multiplies the function *f*. If θ = 1 we had the observed function, but if θ > 1, the demographic process that corresponds to *f* increased. We were able to estimate λ values for any θ and any function *f*. From the equation of the slope, the elasticity *s*_*f*_ of function *f* was calculated as

$$s_{f}=0.5\left(\frac{\ln\lambda_{obs}-\ln\lambda_{1.01f}}{-\ln\left(1.01\right)}+\frac{\ln\lambda_{obs}-\ln\lambda_{0.99f}}{-\ln\left(0.99\right)}\right),$$

where λ_*obs*_ is the population growth rate estimated for θ = 1, and *λ*_0.99*f*_ and *λ*_1.01*f*_ are *λ* estimated after multiplying *f* by θ = 0.99 and θ = 1.01, respectively. The two slopes were averaged (hence the sum and multiplication by 0.5) to obtain a better estimate of the derivative. To understand what this procedure estimates consider, for example, survival: the elasticity of survival is the proportional change in growth rate as a result from proportional changes in the survival of individuals of every possible size.

### Ecological niche modeling

Presence data of *O*. *rastrera* were obtained from the Mexican government biodiversity database (Sistema Nacional de Información sobre Biodiversidad de México) (www.biodiversidad.gob.mx) and validated with help from a taxonomic cacti expert (Dr. Salvador Arias). To reduce bias in the modeling procedure with MaxEnt, we created an overlapping target background group (TGB) with two sister species, *O*. *engelmanni and O*. *lindhemeri* (also validated by Dr. Arias), and used presence records of all three species as background points. We included an overlapping TGB to improve the discrimination capacity of MaxEnt between suitable and unsuitable conditions [27]. The fact that a sister species was recorded in a place and *O*. *rastrera* was not found increases the probability of true absence. MaxEnt develops the niche models, inferring probabilities from presence and background information, meaning that it extracts the environmental conditions where a species has been recorded and from a sample of the rest of the study area (background). One important assumption in this process is that the sampling of the target species and the background were random across the study area. When this is not the case, it is recommended that the background sampling have the same bias as the target species record sampling [28]. For example, it is common that species are recorded along roads; therefore, background sampling should be restricted along roads as well. Ultimately, we gathered 43 presence records of *O*. *rastrera* and 225 background points of both *O*. *engelmanni* and *O*. *lindheimeri*.

To reduce bias in the records due to higher sampling effort in specific areas, we used the R package spThin [29], which randomly eliminates some points that are separated by less than 10 km, with the aim of retaining the largest number of records. This process was repeated 50 times. Final data sets varied between 20 and 30 records and for each repetition we estimated the AUC. The final AUC score was calculated as the mean of the 50 repetitions. We used the ROCR R package to estimate AUC.

We chose the MaxEnt algorithm [27] to model the ecological niche and distribution of *O*. *rastrera* because it has performed well for several species and is a widely used modeling algorithm [20,30,31,32]. We carried out 100 repetitions with a 70:30 random training/testing data partitioning and used the TSS (0.6) evaluation metric to build a mean weighted model ensemble [33] because ensembles have proven to be an effective way to deal with uncertainty [34]. All analyses were carried out in the BIOMOD platform [33]. Although we are aware of the use of ENMval to evaluate models with different settings of parameter combinations using criterion such as the AICc (Akaike Information Criterion) [35], we kept the default parameterization because its predictability performance was better (TSS = 0.629, AUC = 0.861) than the model with the lowest AICc (TSS = O.418, AUC = 0.753).

Niche models were constructed with the four bioclimatic variables that were significant predictors to demographic rates to make it comparable with the climatic-dependent demographic model. Model validation was via the area under the curve (AUC) of the ROC plots [13,25] and the TSS test of the 100 repetitions ensemble. We compared the suitability map resulting from MaxEnt with the demographic model and analyzed the correlation between suitability and *λ*.

Finally, we calculated the distance to the niche centroid to compare these values with the suitability values coming from MaxEnt and *λ*s coming from the climate-dependent demographic model. We calculated the distance to the niche centroid by using the binary maps coming from MaxEnt (probability maps were converted into a presence-absence map using a probability threshold value that minimized both omission and commission errors) and obtained the standardized mean (values that represent the center of the ecological niche) of each of the bioclimatic variables from each pixel that represents presence of the species. Then, we calculated the bioclimatic multidimensional Euclidian distance of each pixel in the study region (with its specific bioclimatic profile) to the standardized mean [4]:

$$d\left(P_{i},N\right)={\left(\sum_{j}{\left(P_{i,j}-N_{j}\right)}^{2}\right)}^{\frac{1}{2}},$$

where *P*_*i*,*j*_ and *N*_*j*_ are the values of the *j*-th bioclimatic variable in pixel *i* and niche centroid, respectively. The smaller the distance to the niche centroid the higher the growth rates expected.

## Results

### Niche distribution modelling with two different approaches

**Fig 1:**
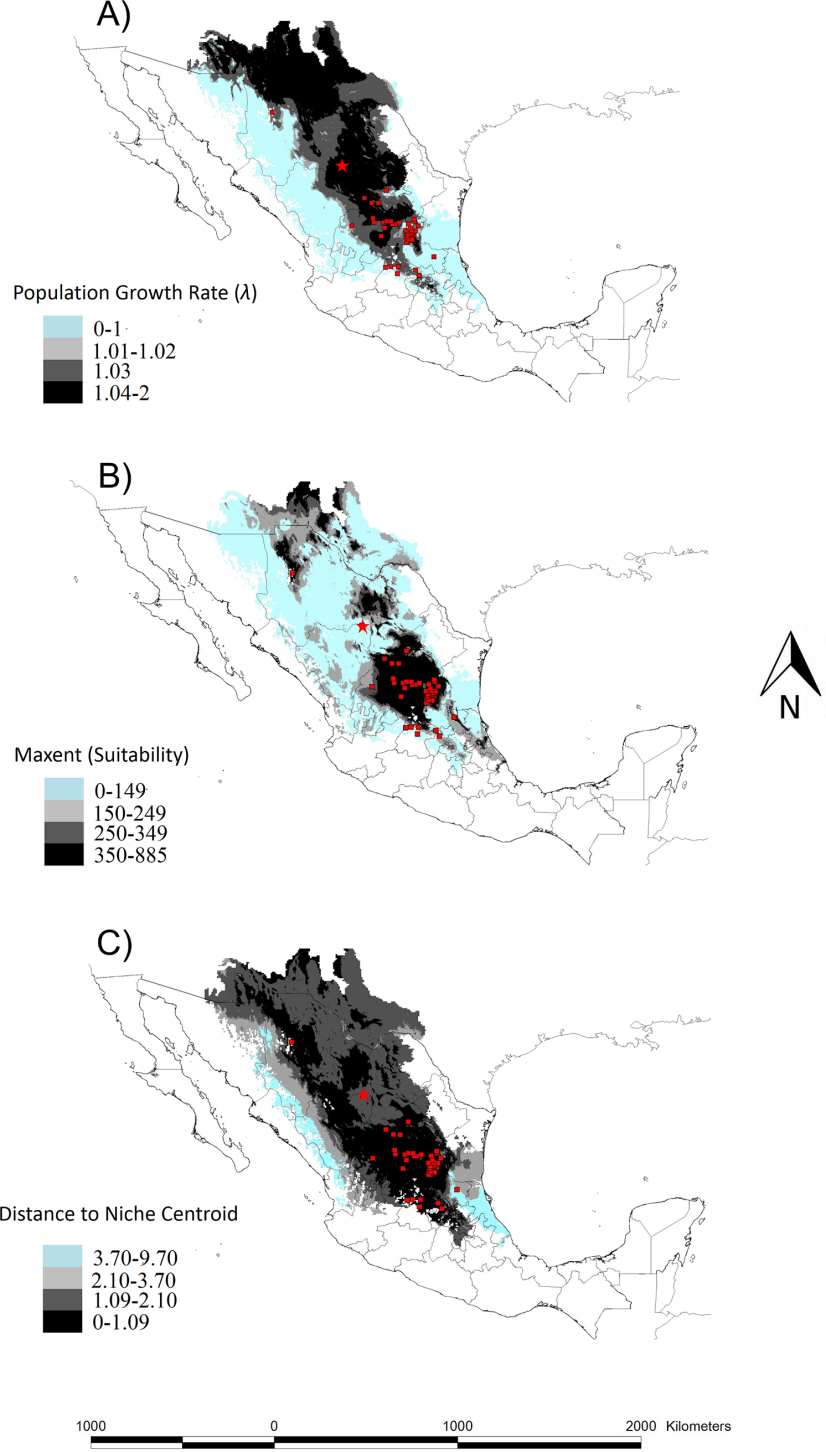
Climate-dependent demographic model, MaxEnt and distance to niche centroid. A) Climate-dependent demographic model. The threshold value *λ* = 1.03 represents the best distribution model from the models with a *λ>*1, B) MaxEnt model. The greater the suitability value, the more appropriate that area is expected to be for the species, C) Distance to niche centroid values. The smaller the distance to the niche centroid, the higher the fitness expected from the species. In the three maps, the site of the demographic study is represented by a star. The sites where the species was recorded are shown as red squares.

MaxEnt predicted a core area of distribution in central Mexico, which encompasses the vast majority of the records of *O*. *rastrera*, with the addition of some small satellite areas scattered to the north. The demographic model with the best possible threshold value was *λ* = 1.03 TSS (0.35) (Fig 1). This binary map and the MaxEnt model provided similar boundaries for the core area, with the exception of the western limits. The demographic model produced a much larger presence area than MaxEnt, and despite the climate-dependent demographic model giving better than random results (AUC = 0.68 when validating the continued map; TSS = 0.35 when validation a binary map), it performed poorly compared with the MaxEnt model (AUC = 0.861, TSS = 0.63).

**Fig 2:**
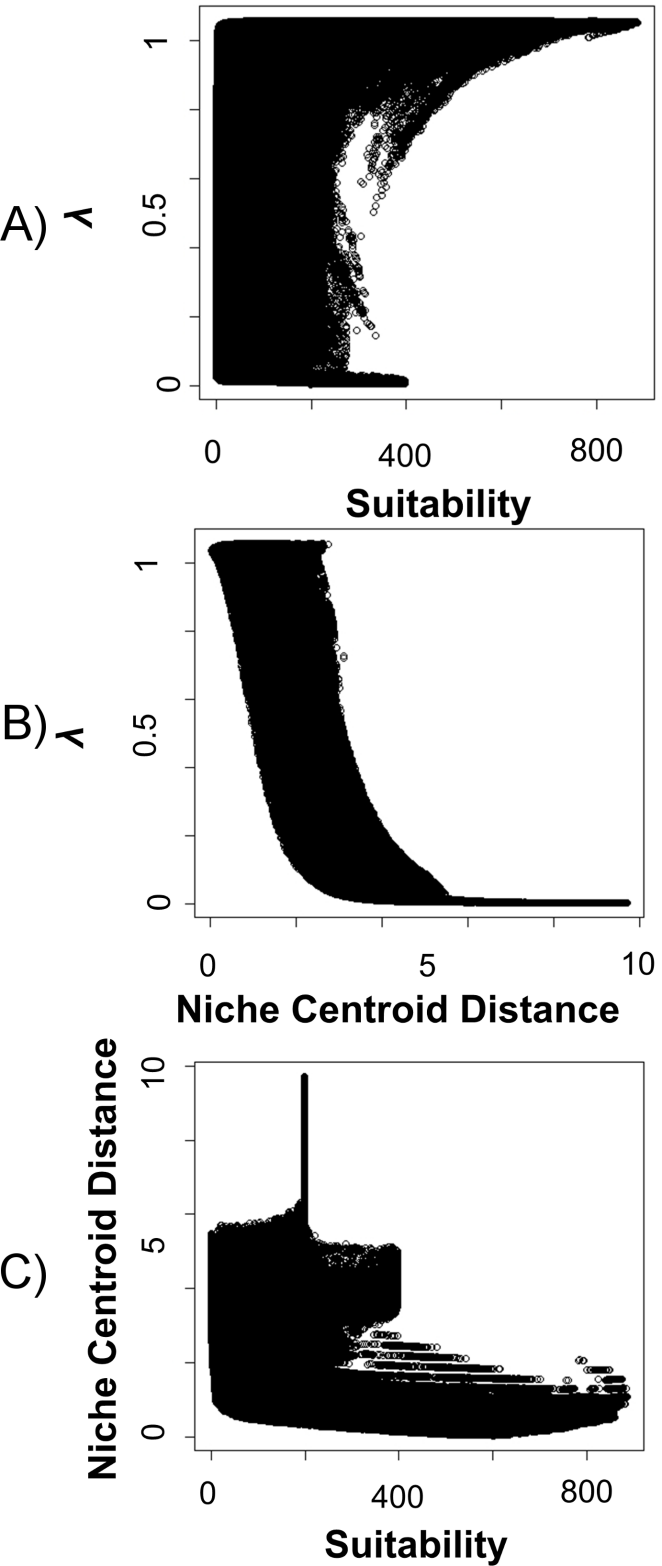
Comparisons between population growth rates, suitability and niche centroid distance. A) Popolution growth rate *vs* MaxEnt suitability values, B) Population growth rate *vs* distance to niche centroid values and C) MaxEnt suitability values *vs* distance to niche centroid. Each circle in the plots corresponds to one of the pixels in the study region.

There was a significant relationship (*P* < 0.0001) (Spearman’s correlation) between *λ*-values and MaxEnt suitability values (Fig 2A). However, an r_s_ = 0.389 indicates that the correlation between *λ* and MaxEnt suitability values is not strong. Instead, the relationship between *λ*-values and the distance to the niche centroid (central values of the ecological niche) was highly significant (*P*< 0.0001) and very robust (Spearman = -0.706) (Fig 2B). Finally, no informative relationship between niche centroid distance and MaxEnt suitability was observed (Fig 2C).

### Climate effects on demography

**Fig 3:**
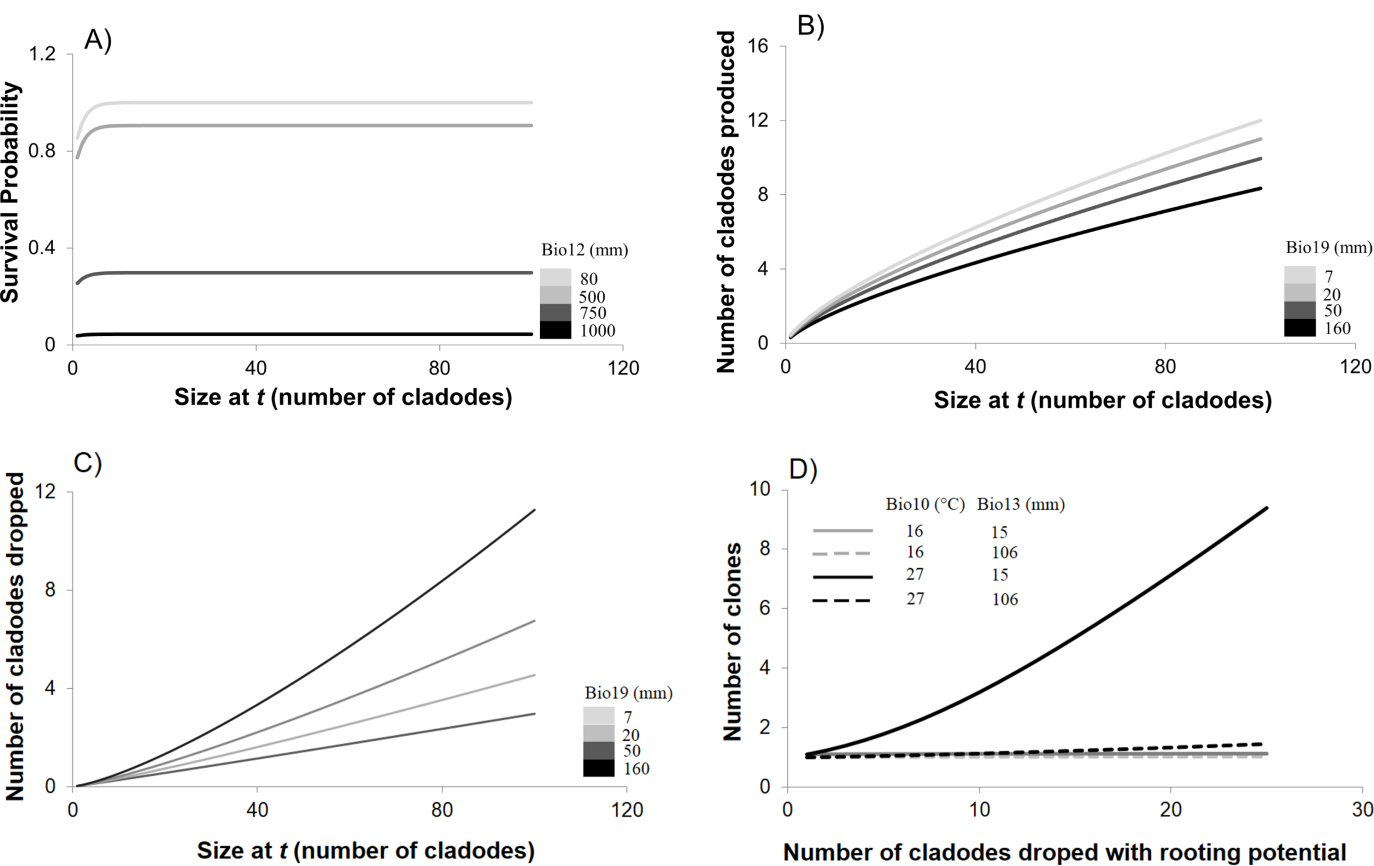
Relationship between vital rates and bioclimatic variables. A) Relationship between survival probability, plant size (in number of cladodes) and annual rainfall (bioclimatic variable Bio12). B) Relationship between the numbers of cladodes produced annually (growth), plant size and the precipitation of the coldest quarter (Bio19). C) Relationship between the numbers of cladodes dropped annually, plant size and the precipitation of the coldest quarter (Bio19). D) Relationship between the number of cladodes that were dropped with rooting potential, plant size and mean temperature of the warmest quarter and precipitation of the wettest month (Bio10 and Bio13, respectively).

Survival was negatively related to annual rainfall (Bio12) (Fig 3A). Similarly, there was a negative relationship between the precipitation of the coldest quarter of the year (Bio19) and the production of new cladodes (growth) (Fig 3B). Instead, the number of cladodes dropped was positively related to the precipitation of the coldest quarter of the year (Bio19) (Fig 3C). Finally, the number of clones into which the cladodes that were dropped and divided was significantly influenced by two bioclimatic variables: (a) the number of clones was negatively related to the precipitation of the wettest month (Bio13) and (b) positively related to the temperature of the warmest quarter (Bio10) (See Fig 3C). For some kernel parameters, the model with the lowest AIC value had biologically unlikely behaviors (for instance, the appearance of immortal individuals) when extrapolated to the whole range of climatic conditions observed in *M*. In such cases, we chose another model from the set of models with ΔAIC < 2 that resulted in biologically more meaningful results. In the cases were the null model had a ΔAIC < 2, we also selected it, as it was more parsimonious. Also, when the AIC diminished in less than two units when a variable was added, it was excluded [36] (S5 File).

To assess how climate determines the geographic variations in population growth, we plotted the *λ* values and MaxEnt suitability obtained for each pixel in the study region with the four informative bioclimatic variables. We also integrated the observed climatic variability in the regular ecological niche approach and in the climatic dependent demographic model approach to evaluate differences between them. The space approach (commonly used for ecological niche modeling) had a larger range of climatic variability for variables Bio 10 (mean temperature of warmest quarter) and Bio 12 (annual precipitation) (18-28ºC *vs* 23-26ºC and 254–1223 mm *vs* 122–374 mm for space and space-time, respectively). However, for variables Bio 13 (precipitation of wettest month) and Bio19 (precipitation of coldest quarter), the time for space range was greater (12–61 mm *vs* 14–124 mm and 33–70 mm *vs* 0–79 mm for space and time, respectively).

**Fig 4:**
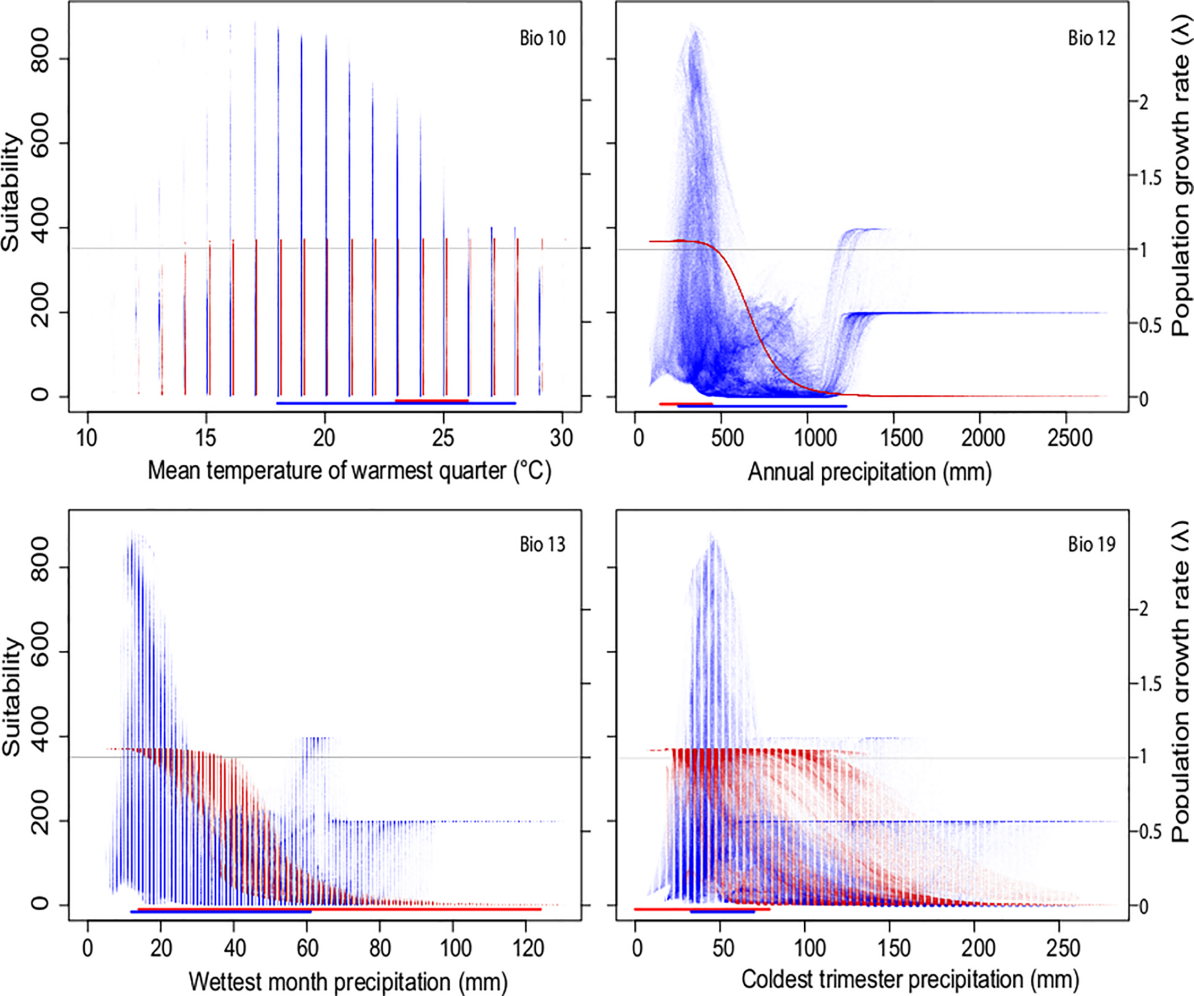
Effects of climate and population growth. Relationships between bioclimatic variables, habitat suitability and population growth rates (*λ*). Points in each graph correspond to each pixel in the study region. Suitability values (blue) were obtained from MaxEnt, and *λ* values (red) from the demographic model. The bars below each graph correspond to the intervals for which time-averaged climatic data were available throughout the region (blue), or the observed temporal variations at Mapimí (red). The grey line corresponds to suitability = 350 and *λ* = 1; below these numbers, *O*. *rastrera* rarely occurred (see Fig 1).

Demographic and niche models indicate that the range of temperature where the species could be distributed is wide (Fig 4). However, in the niche model, suitability values decreased after 25°C, which could not be identified by the demographic model that had a *λ≥*1 in the entire temperature range (10–30°C) *λ≥*1 (Fig 4). Meanwhile, annual precipitation strongly determined *λ*, causing it to decrease (Fig 4). Similarly, suitability decreased importantly after 500 mm of precipitation. Both models reflect a limitation with an important increase of precipitation, but the demographic model failed to predict a limit in water scarcity that the MaxEnt model identified around 250 mm. Because the only vital rate affected by annual precipitation was survival, the observed reductions in *λ* with rainfall could only have been caused by reduced survival. This result is also supported by an elasticity analysis, which showed that by far the vital rate with greatest elasticity is survival (Table 1).

**Table 1:** Elasticity values. We conducted an elasticity analysis by modifying by 10% (1.01), one by one, each of the vital rates in the kernel to see how growth rate behaved with such modifications. Survival: survival probability of a plant having the following: clone number: number of clones produced by a mother plant, growth: probability a plant of producing cladodes after one year, number of cladodes: number of cladodes dropped by a mother plant that established and loss of cladodes: probability of a plant to drop cladodes in one year.

Vital rate	Elasticity
**Survival**	0.8674
**Clone Number**	0.1385
**Growth**	0.0657
**Number of cladodes**	0.0002
**Loss od cladodes**	-0.0001

Precipitation in the wettest month and in the coldest quarter of the year, which influenced growth and fecundity, respectively, also showed an influence on *λ* and suitability values. Precipitation in the wettest month caused an important decrease in suitability values after 38 mm and after 45 mm in *λ* values. Precipitation in the coldest quarter (Fig 3) had a minimal effect on growth rate, but high suitability values were clearly between 25–75 mm (Fig 3).

## Discussion

### Niche distribution modelling with two different approaches

The potential distribution map produced with the climatic-dependent demographic model showed similarities with the MaxEnt model, but the former had a greater commission error. Nevertheless, the climatic-dependent demographic model correctly predicted the presence of *O*. *rastrera* in Mapimí, where the demographic data were collected, whereas the MaxEnt model did not. This may be due to a bias in sampling effort in the southeastern portion of the distribution area, despite efforts to correct for this. This is likely to bias the climate envelope of *O*. *rastrera* generated by the ENM toward conditions prevalent in the intensively sampled area and may also explain why the algorithm failed to assign a high suitability to Mapimí [27].

The basis of our model is the substitution of variation in the climatic variables across space with variability over time, implying important differences compared with studies based purely on spatial variation of climate [5] or ENM algorithms. ENMs indirectly incorporate the effects of other variables that affect the performance of the focal species and that are also correlated with climate [13]. For instance, consider the scenario where *O*. *rastrera* is absent from some climatically appropriate areas but are occupied by some other *Opuntia* species, which are perhaps outcompeting it. Data collected from these sites would suggest that the local conditions are unfavorable for *O*. *rastrera* when in fact the underlying mechanism—competition with a relative—is not apparent. Data from only one site could not be a result of competition because the competing *Opuntia* were not present at the site in favorable years and disappeared during adverse periods. Data collected at one site reflect physiological changes in the study species with climatic conditions and perhaps only represent interactions with short-lived species whose density responds immediately to climatic fluctuations. This can be seen either as a benefit or as a drawback, depending on the aims of the study. If the sole purpose of the work is to predict distribution area of a species, then data collected over different sites incorporating the effect of competitors or predators contains more information and may result in more accurate geographic projections. In contrast, if the study seeks to determine how climatic and other environmental changes influence specific vital rates, abundance or other ways of analyzing fitness, then the follow-up of a single site through time may be more appropriate.

This analysis can also give more information about the fundamental niche of the studied species. Thus, the comparison between models based on spatial and temporal data may be especially informative. Our climate-dependent demographic model may correctly predict the area where *O*. *rastrera* can occur given the local climate, while the MaxEnt model may be cropping this “fundamental niche area” into its smaller areas of “potential realized niche” by indirectly accounting for the presence of predators or competitors. The concurrent use of demography and ENM provides valuable insights into the factors and processes limiting the distribution of *O*. *rastrera*. It is remarkable that the north, east and south boundaries of the core of the potential distribution area predicted by both approaches are almost identical. This strongly suggests that the process determining some of the boundaries of the area projected by ENM are fundamentally determined by a high mortality rate due to excessive precipitation. Moreover, the fact that boundary populations accurately predicted such boundaries suggests that intolerance to high precipitation is a conserved niche attribute in *O*. *rastrera*.

Recent work has proposed that ENM-derived metrics provide valuable information about the underlying population attributes [4,37]. In our work, the relationship between high-suitability areas estimated with MaxEnt and high *λ* values was weak. However, the distance to the niche centroid was a much better approach for representing the spatial variation of population growth rate, proving that it is a good method for estimating population densities or crop productivity [38,39]. This fact has important implications for conservation, especially when time, money and information are scarce. Consequently, rather than only using suitability values from MaxEnt results or other ecological niche algorithms to calculate higher fitness, for example abundance [37], we might calculate the values representing the ecological niche centroid and calculate the environmental distance to it. As tested in this work, higher fitness is expected the closer we are environmentally to these values.

### Climate effects on demography

In deserts, water is the most important limiting factor for plant performance. However, cacti have morphological and physiological traits to withstand water scarcity, such as succulence and CAM photosynthetic metabolism [40]. In *O*. *rastrera*, it seems that high levels of ground or surface water causes hydric stress and higher densities of pathogen fungi in the soil ([41] and personal observation). An excessive intake of water in very rainy years also leads to the collapse of cladodes due to their increase in weight (personal observation). These phenomena have also been seen in other cacti [42]. Furthermore, species of *Opuntia* species have a strong heat resistance [43], tolerating higher temperatures than other species.

Annual precipitation was the determinant factor of *λ*, and the mechanism behind this relations is survival which is the only process affected by annual rainfall. Because the survival of adult plants in most long-lived perennials has a strong influence on the population growth rate [44], we were not surprised to find that survival determined *λ*. It seems that, when facing changes in rainfall, *O*. *rastrera* follows a demographic buffering strategy, i.e., it has the ability to buffer environmental changes by keeping key demographic components unchanged [45]. In *O*. *rastrera*, the survival asymptote was very stable over time (coefficient of variation = 0.025) in comparison with other vital rates that were also influenced by climate (range of coefficients of variation 0.467–21.982). Survival was by far the vital rate with greatest elasticity, and the fact that its response to environmental conditions was less labile than other vital rates suggests demographic buffering. Nonetheless, other vital rates also affected *λ* values under different climatic conditions. Growth, the loss of cladodes and clone numbers depended on timing of precipitation and temperature. However, these bioclimatic variables did not determine the population growth rate as strongly as annual rainfall.

Extensive works have characterized how physiological traits change with drought in cacti [40,46], but further efforts are needed to understand the physiological response to water that may limit population growth. In addition, physiological experiments are needed to gain a better understanding of how vital rates of *O*. *rastrera* are affected by changes in climatic variables. Information obtained from such experiments would enrich demographic and ecological niche models.

### Model limitations

The most common ENM technique is to relate geographic records with environmental layers and create an environmental profile although other approaches may be based on physiological [47] or demographic [5] data. Undoubtedly, the assumptions behind the models are key aspects for the differences among models. The use of temporal variation in vital rates to determine geographic ranges is based on a number of assumptions. First, the species’ vital rates change with climate in the same way across the species range. This assumption may be justified if niche is in fact conserved in related taxa [48] because we would then expect it to be quite similar among populations of the same species. Nevertheless, adaptations to local climate [49] would potentially cause our method to fail. A second assumption is that plants respond to long-term climate in the same way as to short-term fluctuations. The capability of individuals to buffer short-term conditions may cause this assumption to fail. For instance, some species can survive a year without rainfall because they can store water (hence buffering drought), but they would certainly die in a site with a long-term mean precipitation of zero. In contrast, other events may have effects on the life cycle, even in the short term. This is the case of the death of *O*. *rastrera* individuals that absorb too much water in rainy years. In such cases, a single short-term event may accurately represent what would happen in a site with a high long-term precipitation. Perhaps this is why our climate-dependent demographic model was able to explain why *O*. *rastrera* is unable to colonize rainy areas but failed to identify the boundaries of its distribution toward low-precipitation regions. A third assumption is that the time-averaged population transition model (like the one that we estimated for the mean climatic conditions in each pixel of *M*) accurately represents the growth rate of the population in a fluctuating environment. Previous studies have shown that this is the case for *O*. *ratrera* [11]. However, a common consequence of environmental fluctuations on population dynamics is a reduction in *λ* compared to that estimated assuming a constant environment. This may be the reason why a threshold of *λ* = 1.03 produced a better estimate of *O*. *rastrera’s* distribution area than *λ* = 1, which would suffice for population subsistence in the absence of fluctuations. The fourth assumption is that factors, such as biological interactions that may affect population growth rate, respond very rapidly to climatic fluctuations. This is unlikely to occur in many cases. Consider the case where a natural predator limits the distribution of *O*. *rastrera* in warm regions. If Mapimí is outside the distribution range of this predator, it will not appear suddenly in Mapimí in warm years, and thus the demographic model would be unable to predict that *O*. *rastrera* does not occur in areas with high temperatures. Another important limitation of our model is that relationship between climatic variables and demographic parameters was not linear and extrapolation becomes difficult and dependent on the function form. This challenge might be resolved by only extrapolating in pixels that are within the range of climatic variables.

## Implications

Here, we have shown that detailed, long-term demographic studies, even at a small-scale, might help to answer the mechanisms behind geographic distributions. ENM may be more accurate when predicting the potential-realized niche distribution than models based on demography, but demographic models give biological insights of why the species is being distributed as it is. Our results show a low correlation between climatically suitable areas projected by MaxEnt and higher population growth rates. This suggests that higher suitability not necessarily reflects higher species fitness. Instead, we found a significant and robust correlation between environmental distance to the niche centroid (center of the niche hypervolume) and population growth rate. Consequently, the niche centroid approach might be helpful in finding higher fitness areas in the geographic distribution of other species. Finally, we would like to highlight that the development of hybrid methods may take advantage of the assets of both approaches resulting in more accurate and meaningful results.

## Supporting information

S1 File Demographic model. **Table A Number of individuals and clones.** This table shows the number of individuals and clones registered each year of the demographic study. **Figure A Comparison of the observed size distribution (black bars = and that projected by the demographic model (red line).**
(DOCX)

S2 File “R” code. (TXT)

S3 File Model selection statistics. The best models are shown in red. The model with lowest AIC is shown in bold characters, while the next best models (DAIC <2) are shown in italics. The model that was selected for simulating population dynamics is shown with a yellow background. This model was selected based on two criteria: First, the sum of the AIC values of all years (excluding years for which there were convergence problems or no data), generates the AIC for any given model; this should be the lowest in the model set, or have a DAIC < 2. Second, the model should be the best (or indistinguishable from the best) in the largest number of years. **Table A. AIC values for survival models** The null model corresponds to a constant survival probability, whereas in the other models, survival was a function of the initial number of cladodes *x*. Values shown correspond to different curves with an upper asymptote different from one. We tested logistic and complementary log-log functions. In all cases the error was binomial. No mortality was observed in 1992. **Table B. AIC values for plant growth models** In all cases, the final size of a plant is assumed to be equal to its initial size *x*, plus the number of cladodes produced *b*(*x*) and minus the number of cladodes dead or dropped *d*(*x*). Two sets of models were tested. In one of them, the numbers of cladodes produced or dead is a linear function of size (e.g., *b*(*x*) = *a*_0_+*a*_1_*x*), and in the other, the function is nonlinear (exponential) of size *b*(*x*) = exp(*a*_0_+*a*_1_*x*). Preliminary exploration of the data revealed that these two options produced the best fits. In both cases, the null model corresponds to the case where the numbers of neither produced nor dead depend on x. The models indicated as *d*(*x*) and *b*(*x*) correspond to the cases where only the number of dead or produced cladodes depended on *x*, respectively. Finally, *d*(*x*) + *b*(*x*) indicate the models where both processes depend on the initial size. Cladode death and production each have their own distribution and are summed to obtain the final size of each plant. This is why the distribution of the latter is a convolution of two distributions. In the table, the first distribution corresponds to cladode production and the second to cladode death. Some distributions are truncated because at most *x* cladodes can die. Models with convergence problems are shown as NA. **Table C. AIC values for fecundity (number of cladodes) models** A large number of individuals did not reproduce; this is why the appropriate distribution for the number of cladodes that were dropped and became established was suspected to have a substantial inflation in zero. After exploring Poisson, negative binomial and zero inflated Poisson, we chose a zero inflated negative binomial distribution because of its substantially smaller AIC values. All models shown here assume such distribution. The model comprises two components: a model for the (uninflated) negative binomial distribution's mean, and a second model for the zero inflation factor. Both of these models may be, respectively, exponential or logistic functions of different explanatory factors: the number of cladodes in the mother plant at time *t*, *x*, and at time *t* + 1, *y*. Null models are those where the mean or inflation factor do not depend on either of the explanatory variables. NA indicates models in which convergence was doubtful. **Table D. AIC values for fecundity (number of clones) values** Once a plant has dropped a number of cladodes that become established, these may be arranged in one or more clones. Thus, the error distribution had to be zero truncated. Exploratory analyses showed that a zero-truncated negative binomial produced much larger AIC values than a zero-truncated Poisson model; thus, we chose the latter to model the number of clones produced by a mother plant. We used the size of the mother plant at times *t* and *t* + 1 (*x* and *y*, respectively) and *m*, the number of cladodes that are dropped and become established, as explanatory variables.
(DOCX)

S4 File Demographic parameters. **Table A.** Parameters for survival and growth functions. **Table B.** Parameters for fecundity functions. No reproduction was recorded in the years for which there are no parameters.
(DOCX)

S5 File Data for the selection of the models that link demographic parameters to bioclimatic variables. Three models were tested in every case: ID, a linear model relating the parameters with the climatic variables without any transformation; Log, as before, but log-transforming the bioclimatic variable; and Inv, in which the reciprocal (multiplicative inverse) of the bioclimatic variable was used. The model with the lowest AIC was selected and the function plotted over the whole range of climatic parameters observed in the study region. If the function showed an unusual behavior when extrapolated to the whole region (e.g., it resulted in biologically absurd parameters), we fitted two new models suggested by the form of the relationships observed: LogInv, in which the bioclimatic variables where log-transformed and their inverse calculated; and InvLogInv, which was as LogInv, but with an inverse link function. This procedure was only performed for the bioclimatic variable with the lowest AIC. We selected the function that was most similar to the one with the lowest AIC over the observed range of the bioclimatic variable but that produced biologically sensible estimates when extrapolated. In all the following tables we follow the next conventions: *Numbers highlighted with yellow*: Model used, *Red numbers*: Do not differ from the best model, *Bold numbers in red*: Best model but not necessarily the model used depending on AIC differences and following parsimony. For parameter *θ*_b_, we kept the variable with greatest influence but used a different model than the one with lowest AIC because it resulted in biologically more meaningful results when extrapolating. For parameters *α*_i_, the model with the lowest AIC differ minimally from the null model (ΔAIC < 2) and for parsimony, we kept the null model. In the case of *β*m, the ΔAIC value between the null model and the best one was 2.03, but we still selected the null model. This was because the best model for *β*m implied the inclusion of the bioclimatic variable bio14 in the analyses thus increasing the computational time required to produce the kernels for each pixel in the map by months. **Table A.** AIC values of the models that relate survival parameters with the selected bioclimatic variables. **Table B.** AIC values of the models that relate growth parameters with the selected bioclimatic variables. **Table C.** AIC values of the models that relate number of cladodes dropped or established parameters with the selected bioclimatic variables. **Table D**. AIC values of the models that relate parameters of clone production with the selected bioclimatic variables.
(DOCX)

S6 File Demographic data. (XLSX)
