# Supplementary material for: Inferring space from time: On the relationship between demography and environmental suitability in the desert plant O. rastrera
Source: PLoS One. 2018 Aug 9;13(8):e0201543. doi: 10.1371/journal.pone.0201543 (PMC6084933; doi:10.1371/journal.pone.0201543)
Supplement: S1 File — Table A Number of individuals and clones. This table shows the number of individuals and clones registered each year of the demographic study. Figure A Comparison of the observed size distribution (black bars = and that projected by the demographic model (red line). (DOCX) [file pone.0201543.s001.docx]

**S1**. **Demographic model**

**General structure of the model**

Demography projects the consequences of the life history traits of individuals and their variability to whole populations. Because such traits depend usually on the individual’s status, many demographic models are structured either by age, size, or any other variable that determines the organism’s behavior. By far, the most popular tools in ecological studies of structured populations are matrix projection models (MPM) and integral projection models (IPM). MPMs rely on a categorization of individuals into ς discrete classes. The demographic processes are then incorporated into a ς × ς matrix **A** that projects the transitions of individuals from time *t* to time *t* + 1 through the equation **n***_t_*_+1_ = **An***_t_*, where **n***_t_* is a vector whose elements are the number of individuals in each category at time *t*. IPMs are widely used when the variable *x* that describes the status of an individual al time *t* is continuous. Instead of a matrix, IPMs summarize the demographic processes in a function *k*(*x,y*) known as the kernel, where *y* is the status of the individual at time *t* + 1. The kernel is usually composed of individual functions that describe survival, growth and reproduction. The projection of the population over time is achieved through the equation (hence the “integral” in IPM). The functions *n* in this model consist in a probability density function that describes the population structure multiplied by the population size. Thus, the integral of *n* over the whole interval **Ω** of individual sizes is the population size.

In plants, size has proved to be a major determinant of the main demographic processes that influence population dynamics: survival, growth and reproduction. In *Opuntia*, the most natural measure of size is the number of cladodes. This is a discrete variable suitable for MPMs. However, IPMs have a number of characteristics that are advantageous for our study: (1) demographic processes are modeled as functions, so weather variables are statistically and conceptually easy to incorporate into the functions. (2) By keeping each demographic process separate from the others in the kernel, we have explicit information on the components of composite entries (e.g., matrix elements that combine retrogression and vegetative propagation, the main form of reproduction in *Opuntia*) that frequently occur in MPMs. Also, all the non-fecundity entries in a column of **A** add up to survival. These assets resolve some complications that introduce non-zero covariances between matrix entries, and that would be difficult to model if MPM’s were used. (3) If the number of categories in **A** is large enough to avoid misrepresentation of the individuals within it, an inordinately large dataset is required to estimate accurately every entry in the matrix.

Thus, we used a hybrid model: to account for the discrete nature of our size variable, we use a 100 × 100 projection matrix **K** because *Opuntia rastrera* individuals rarely have more than 100 cladodes. Each row and column in the matrix corresponds to an exact number of cladodes, avoiding arbitrary categorizations. However, the entries of **K** were not estimated from conventional methods where transitions in the matrix estimated from the observed fraction of individuals that transit between size categories (Caswell 2001), but obtained from a kernel *k*(*x,y*) as in IPMs. Unlike traditional IPMs, our kernel was based on discrete probability distributions and not on probability density functions.

Because the distribution of a species will depend on its population growth rate in different areas, we required an accurate estimation of this parameter. It has been shown that the selection of the functions that comprise the kernel is critical for this purpose, with poor choices leading to incorrect estimations (Dahlgren et al. 2011). Thus, were fitted a large number of alternative functions to the demographic data for each year individually. Unless otherwise stated, we used the package bbmle for R for this purpose. The AIC of each model was used to select the best one. When the model with the lowest AIC differed between years, we chose the one that was the best in the most cases (See Table 1 for number of individuals and clones used to estimate **K**).

In the following sections we describe the functions that were selected for each demographic process. See Appendix 2 for the statistics on model selection. Parameters that were estimated from the data are represented by Greek letters, whereas the Latin alphabet is reserved for variables and functions.

**S1 Table A**

| **Year** | **92** | **93** | **94** | **95** | **96** | **97** | **98** | **99** |
| --- | --- | --- | --- | --- | --- | --- | --- | --- |
| Individuals | 553 | 592 | 590 | 589 | 589 | 569 | 809 | 747 |
| Clones | 0 | 0 | 44 | 0 | 0 | 148 | 0 | 52 |
| **Year** | **2000** | **2001** | **2002** | **2003** | **2004** | **2005** | **2006** | **2007** |
| Individuals | 707 | 722 | 884 | 909 | 1122 | 1047 | 1038 | 1138 |
| Clones | 40 | 36 | 36 | 33 | 30 | 0 | 72 | 41 |

**Demographic-process functions**

*Survival and growth*

The best function for modeling survival depending on size *x* at time *t* was a logistic with an asymptote *γ_s_* different from one

. (1)

In general, the size *y* at time *t* + 1 was similar to *x*, but the distribution of *y* had a very long tail for *y* < *x*, and a short one for *y* > *x*, for which we did not find any simple formula. Such skewed distribution probably reflects the fact that individuals undergo both production of new cladodes *b* and death of old ones *d* simultaneously. These two processes were found to depend on plant size, so we modeled them as *b*(*x*) and *d*(*x*), respectively. Thus, the final size may be described as

*y* = *x* + *b*(*x*) –*d*(*x*). (2)

The observed skewness in the distribution of *y* may arise if individuals experienced variable decreases in their size by means of dropping capricious numbers of cladodes, whereas the number of cladodes produced was likely to be more constant. Thus, equation 2 was used to model growth. Because we did not have data for the cladode production and dropping, we tried different functions and error distributions for *b*(*x*) and *d*(*x*) and chose the ones that predicted *y* better. Because *y* is a sum of two random variables, likelihood (and thus AIC) was computed from the convolution of the two underlying distributions.

The chosen model for the distribution of *b*(*x*) was a negative binomial with overdispersion parameter *θ_b_*, and for *d*(*x*) a negative binomial truncated at *x* – 1 (because no plant cannot drop all its cladodes, which would result in death, or more cladodes than it has) with overdispersion parameter *θ_d_.* The following functions were the best for the means and of *b*(*x*) and *d*(*x*) for any given *x*:

(3)

Thus, the probability *g_y,x_* that a plant with a size *x* has a size *y* at time *t* + 1 given that it survives was calculated from the convolution:

, (4)

where Γ is the gamma function. Let **G** be the matrix containing the probabilities *g_y,x_* that a plant with a size *x* has a size *y* at time *t* + 1 given that it survives. The values of *g_y,x_* are obtained simply by evaluating equation (4) at the values of *x* and *y* that correspond to each row and column of **G**, and standardizing its columns to one to avoid unaccounted mortality in the model. Let **P** be the matrix of probabilities *p_y,x_* that a plant with *x* cladodes at time *t* survives and has *y* cladodes at time *t* + 1. Also, let *s_x_*, the survival probability of an individual of size *x*, be value of function *s*(*x*) evaluated at *x*. The entries of **P** were estimated as

. (5)

*Vegetative propagation*

After revising a large number of alternatives we found that the best strategy to model propagation was a three step procedure. First, we estimated *m*, the total number of cladodes that were dropped by a mother plant and became established. Second, given *m*, we developed a model for *c*, the number of different clones produced from those cladodes. Third, we used a probability distribution to assign the *m* available cladodes to the *c* clones.

We found that *m* depended on *x* and *y.* the probability distribution *M*(*m|x,y*) that best described the behavior of *m* was a zero-inflated negative binomial distribution with mean

, (6)

inflation parameter *i*(*x,y*)

, (7)

and dispersion parameter *θ_m._*

Model fitting was performed using package pscl for R (Zeileis et al. 2008). Note that *M*(*m|x,y*) provides estimates of the probabilities that any individual with initial size *x* and final size *y* drops *m* cladodes that become established, including the case for which *m* = 0, *i.e.*, the probability that the individual does not propagate.

We then regressed the number of clones produced on the observed total number of dropped cladodes that got established using only data from cacti that propagated. Thus, the error distribution had to exclude zero. The selected probability function for *c*, *C*(*c*|*m*), was a zero-truncated Poisson distribution with parameter *λ*(*m*)

(8)

The fitting procedure was performed using package VGAM for R (Yee 2010). *C*(*c*|*m*) provides the probability that *c* clones are produced given that there are *m* available cladodes. Note that, because of the truncation, the mean is not *λ*(*m*) as in an ordinary Poisson distribution, but instead it needs to be calculated as

. (9)

A further consideration is that, because no plant can drop more cladodes than it has (that is, *c* ≤ *m*), in the model we set *C*(*c>m*|*m*) = 0. This affects the mean of the distribution that is actually represented in the kernel, and thus a correction is needed (see below).

Finally, we required to assign the *m* cladodes to the *c* clones. Let *q_l_* be the fraction of the *m* cladodes that ends up as part of the *l*^th^ clone. Then, the number of cladodes assigned to the *l*^th^ clone is simply *q_l_* × *m.* Note that the sum of the *q_l_* must equal one. One way to generate *q_l_* values such that 0 ≤ *q_l_* ≤1 and is to use a probability distribution, *F*(*l*|*m,c*). Note that the appropriate choice for *F*(*l*|*m,c*) must be bounded between 1 and *c* because we need probabilities for *c* cladodes. We tested different distributions, fitting them through maximum likelihood to the observed data assuming a Poisson error using package bbmle. The choice for *F*(*l*|*m,c*) was a geometric distribution, which was not the best but did not differ from the best one (i.e., ΔAIC < 2) on most years. The bounded geometric distribution has an appealing property: let π be the parameter of the geometric distribution *F*(*l*|*m,c*), and *L* be a random variable so distributed. Considering the bounds,

. (10)

If *π* = 0, Pr(*L* = *l*) =1/*c,*  and the geometric becomes the uniform distribution. Note that if *π* = 0 and *c* = *m*, then Pr(*L* = *l*)*m* = 1. This is important because if *c* = *m,* each clone is expected to have one cladode, so the logic demands that *π =*0. Thus, we tried some functions to model *π* as a function of *c* that fulfilled the requisite that *π* = 0 when *c* = *m*. The one with the best fit was

. (11)

and thus, the number of cladodes *y_l_*′ in the *l*^th^ clone is

(12)

Rounding is required because the number of cladodes needs to be an integer. Note that, for clarity in the argument that follows, we will use the prime (') to distinguish the size of clones produced between times *t* and *t* + 1, *y*', from *y*, the size of the mother plant at time *t* + 1.

Unlike all the previous equations, which were fitted using the data for each year separately, eqn. (11) was obtained after pooling the data for all years because for some of them we did not have enough data to obtain a reliable estimate of *α_f_*.

All these equations were used to estimate the entries of the matrix **F** whose entries, *f_y,x_* represent the average number of clones with *y* cladodes that an *x*-sized mother plant is expected to produce. This was done in several steps. (i) From equation (12), for every possible combination of *m* and *c* we estimated the number of cladodes in every clone. (ii) From these values, the expected number of clones with *y*' cladodes produced by a mother plant given *m,* $\bar{f}\left(y'|m\right)$was calculated as

$\bar{f}\left(y'|m\right)=\sum_{w} C\left(c_{w}|m\right)\frac{\mathrm{E}C\left(c|m\right)}{\sum_{j=1}^{m} C\left(c=j|m\right)j}$ (13)

where *w* represents the subset of the clones obtained in step (i) that had *y*′ cladodes. It must be noted that because *w* may contain several clones that have the same probability of being produced, the multiplication of probabilities *C* and random variables involved in the estimation of a mathematical expectation is implicit in the sum$\sum_{w} C\left(c_{w}|m\right)$, and in principle it should equal the mathematical expectation. However, it falls short of it because we set *C*(*c>m*|*m*) = 0. The ratio in the right corrects this problem, because the numerator is the actual number of clones expected (eq. 9), and the denominator is the total number of clones produced after truncation. In other words, the correction factor in the right of the formula insures that $\sum_{j=1}^{m} \overline{f}\left(y'=j|m\right)=\mathrm{E}C(c|m)$, avoiding any reduction in fecundity due to the truncation.

(iii) For any given combination of *x* and *y*, we calculated *M*(*m*|*x,y*) and $\overline{f}\left(y'|m\right)$as explained above. From the formula for the mathematical expectation, the average number of clones with *y*′ cladodes produced by a mother plant having initial and final sizes *x* and *y* is

$\overline{f}\left(y'|x,y\right)=\sum_{m} f\left(y'|m\right)M\left(m|x,y\right)$ (14)

(iv) Finally, we need to express fecundity as an average number of clones with size *y′* produced by a mother plant with initial size x. Applying again the formula for mathematical expectation, this is

(15)

It must be noted that *y′*  is a size at time *t* + 1, so at this point we can replace *y′*  by *y*, that is, *f_y,x_* =. The transition matrix encompassing growth, survival and fecundity is then

**K** = **P** + **F**. (16)

*Model criticism*

A way to examine if the demographic model describes the population dynamics accurately is to check if its results make sense biologically and agree with observations. To do so, we calculated the matrices **K** for every year for which we had field data, and iterated the equation **n***_t_*_+1_ = **Kn***_t_* 10 000 times choosing one **K** randomly in each iteration. This is a stochastic matrix model. We dropped the first 1000 iterations to let the system reach its stationary state. We then calculated the annual growth rate *λ_t+1_* as

and obtained the long term stochastic population growth rate *λ_s_* as the geometric mean of *λ_t_* _+ 1_. We also recorded **n***_t_* _+ 1_ for each iteration, and standardized them so that each adds up to one. We then estimated **w**, the mean of the stationary size structure of the population as the arithmetic mean of the standardized **n***_t_* _+ 1_ vectors.

The estimated *λ_s_* = 1.005, very close to the stable value of one expected for natural, undisturbed populations. Moreover, there was a close match between **w** and the observed structure of the population (Figure S1.1). This suggests that our model correctly describes the dynamics of the population at the study site.

**S1 Figure A**


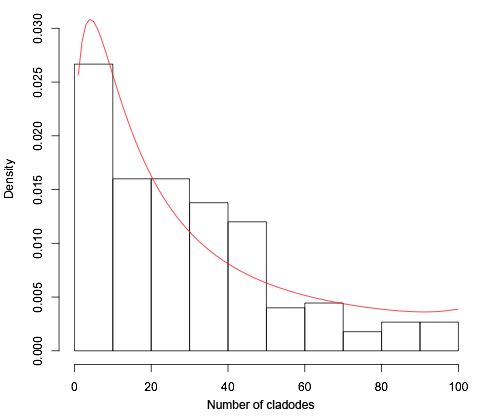


**Effects of climate on demography**

In equations 1, 3, 4, 6, 7 and 8, two or more parameters are estimated from a regression for every year. Thus, the estimated parameters in each model have both a standard error and a covariance. Perhaps the best way to relate them to climate would be to perform multivariate regressions of all the parameters in a model simultaneously on annual climatic data, considering for the variance-covariance matrix of the fitted model. This was done using package mvmeta (Gasparrini et al. 2012) for R. Probably because of the large number of parameters that such method estimates, this procedure resulted in overfitted models that misrepresented the observed processes. Thus, we resorted to univariate regressions of each parameter separately on the 8 bioclimatic variables selected (see main text). Because we had data for only 16 years, we considered that we had insufficient information to include more than one bioclimatic variables in each regression. Thus, for each parameter we performed a set of regressions including only one bioclimatic variable as explanatory variable, plus a null model. Each set was repeated using different regression models, using combinations of logs and reciprocals (multiplicative inverses) of the explanatory and response variables. The model with the lowest AIC was then selected (Appendix 4).

**References**

Gasparrini A, Armstrong B, Kenward MG. Multivariate meta-analysis for non-linear and other multi-parameter associations. Statistics in Medicine. 2012; 31: 3821-3839.

Caswell, H. Matrix Population Models: Construction, Analysis and Interpretation. Sunderland, Massachusetts, U.S.A.; 2001.

Dahlgren JP, García MB, Ehrlén J. Nonlinear relationships between vital rates and state variables in demographic models. Ecology. 2011; 92: 1181-1187.

Zeileis A, Kleiber C, Jackman S. Regression models for count data in R.

Yee, T. W. The VGAM package for categorical data analysis. Journal of Statistical Software. 2007; 32: 1-34.
