# Supplementary material for: Inferring space from time: On the relationship between demography and environmental suitability in the desert plant O. rastrera
Source: PLoS One. 2018 Aug 9;13(8):e0201543. doi: 10.1371/journal.pone.0201543 (PMC6084933; doi:10.1371/journal.pone.0201543)
Supplement: S3 File — The best models are shown in red. The model with lowest AIC is shown in bold characters, while the next best models (DAIC <2) are shown in italics. The model that was selected for simulating population dynamics is shown with a yellow background. This model was selected based on two criteria: First, the sum of the AIC values of all years (excluding years for which there were convergence problems or no data), generates the AIC for any given model; this should be the lowest in the model set, or have a DAIC < 2. Second, the model should be the best (or indistinguishable from the best) in the largest number of years. Table A. AIC values for survival models The null model corresponds to a constant survival probability, whereas in the other models, survival was a function of the initial number of cladodes x. Values shown correspond to different curves with an upper asymptote different from one. We tested logistic and complementary log-log functions. In all cases the error was binomial. No mortality was observed in 1992. Table B. AIC values for plant growth models In all cases, the final size of a plant is assumed to be equal to its initial size x, plus the number of cladodes produced b(x) and minus the number of cladodes dead or dropped d(x). Two sets of models were tested. In one of them, the numbers of cladodes produced or dead is a linear function of size (e.g., b(x) = a0+a1x), and in the other, the function is nonlinear (exponential) of size b(x) = exp(a0+a1x). Preliminary exploration of the data revealed that these two options produced the best fits. In both cases, the null model corresponds to the case where the numbers of neither produced nor dead depend on x. The models indicated as d(x) and b(x) correspond to the cases where only the number of dead or produced cladodes depended on x, respectively. Finally, d(x) + b(x) indicate the models where both processes depend on the initial size. Cladode death and production each have their own distribution and are summed to [file pone.0201543.s003.docx]

**S3 Table A**

**S3 Table B**

**S3 Table C**

**S3 Table D**
