# Supplementary material for: Inferring space from time: On the relationship between demography and environmental suitability in the desert plant O. rastrera
Source: PLoS One. 2018 Aug 9;13(8):e0201543. doi: 10.1371/journal.pone.0201543 (PMC6084933; doi:10.1371/journal.pone.0201543)
Supplement: S4 File — Table A. Parameters for survival and growth functions. Table B. Parameters for fecundity functions. No reproduction was recorded in the years for which there are no parameters. (DOCX) [file pone.0201543.s004.docx]

**S4. Demographic parameters**

**S4 Table A**

| **YEARS** | ***α_s_*** | ***β_s_*** | ***γ_s_*** | ***α_b_*** | ***β_b_*** | ***θ_b_*** | ***α_d_*** | ***β_d_*** | ***θ_d_*** |
| --- | --- | --- | --- | --- | --- | --- | --- | --- | --- |
| 1992 | NA | NA | NA | -0.8368 | 1.0161 | 3.5144 | -10.5251 | -10.4644 | 1.4931 |
| 1993 | 1.8578 | 0.5992 | 1.0000 | -0.2076 | 0.8296 | 1.4938 | -0.1726 | 0.7199 | 0.3727 |
| 1994 | -2.3330 | 2.0190 | 0.9813 | -1.2185 | 0.8229 | 1.3574 | -1.3963 | 0.5874 | 0.2375 |
| 1995 | 1.2428 | 0.4759 | 1.0000 | -1.1917 | 0.7512 | 1.4202 | -1.4860 | 0.6260 | 0.1948 |
| 1996 | 1.2876 | 0.4674 | 1.0000 | -1.1341 | 0.7551 | 1.5896 | -1.2590 | 0.5648 | 0.2684 |
| 1997 | -0.1404 | 0.8343 | 1.0000 | -1.3327 | 0.8650 | 2.2919 | -3.1087 | 1.2519 | 0.3157 |
| 1998 | 0.8681 | 0.5851 | 1.0000 | -3.4942 | 0.7896 | 0.7090 | -2.2753 | 0.6789 | 0.2093 |
| 1999 | 0.2785 | 0.6284 | 1.0000 | -2.9369 | 0.7473 | 0.6556 | -2.9174 | 0.9450 | 0.2093 |
| 2000 | -0.7505 | 1.1976 | 0.9963 | -2.8807 | 0.8413 | 1.1095 | -3.2020 | 1.0061 | 0.2764 |
| 2001 | 2.9472 | 0.0480 | 0.9878 | -1.0787 | 0.8351 | 1.2140 | -2.1381 | 3.1518 | 0.0019 |
| 2002 | 2.3220 | 0.1646 | 0.9969 | -0.5391 | 0.3662 | 0.3083 | -2.5382 | 1.3972 | 0.4810 |
| 2003 | 1.1504 | 0.4545 | 0.9860 | -0.9765 | 0.5152 | 0.7564 | -2.8387 | 1.4384 | 0.2321 |
| 2004 | -0.2613 | 0.4055 | 0.8998 | -0.5457 | 0.7101 | 1.2496 | -3.1197 | 2.4412 | 0.4242 |
| 2005 | 1.8146 | 0.3369 | 0.9950 | 0.2444 | 0.1753 | 0.5915 | -1.5870 | 0.9453 | 0.4614 |
| 2006 | 3.3942 | 0.2417 | 1.0000 | -4.9119 | 0.8475 | 0.3853 | -8.3978 | -61.8100 | 0.0000 |
| 2007 | 4.0594 | 0.2598 | 0.9966 | -1.2400 | 0.8480 | 1.0293 | -12.2000 | 1.3380 | 2.3923 |

**S4 Table B**

|  |  |  |  |  |  |  |  |  |  |
| --- | --- | --- | --- | --- | --- | --- | --- | --- | --- |
| **YEARS** | ***α_m_*** | ***β_m_*** | ***θ_m_*** | ***α_i_*** | ***β_i_*** | ***γ_i_*** | ***α_c_*** | ***β_c_*** | ***α_f_*** |
| 1994 | 0.9896 | -0.7155 | 0.0142 | 69.7200 | -198.600 | 130.4100 | -2.3943 | 1.1323 | 0.8117 |
| 1997 | 0.2767 | 0.4896 | 0.5338 | 1.6775 | -0.6816 | 0.4819 | -1.4580 | 0.7486 | 0.8117 |
| 1999 | -1.2136 | 0.7733 | 0.4824 | 4.2240 | -0.3765 | -0.2505 | -1.4713 | 0.7187 | 0.8117 |
| 2000 | -2.8849 | 1.0428 | 0.1930 | 3.7160 | -18.7200 | 18.2440 | -1.0224 | 0.3040 | 0.8117 |
| 2001 | 0.3511 | 0.4651 | 2.3450 | 6.4693 | 0.1832 | -1.2262 | -4.0919 | 1.7312 | 0.8117 |
| 2002 | 0.1455 | 0.4854 | 2.0872 | 7.2256 | -2.0801 | 0.9849 | -4.5431 | 1.9564 | 0.8117 |
| 2003 | 0.2007 | 0.3168 | 1.9854 | 7.4478 | -1.7319 | 0.4503 | -3.0720 | 1.4430 | 0.8117 |
| 2004 | -4.1550 | 0.8494 | 0.0078 | 6.5890 | -23.5500 | -18.2940 | -2.2573 | 0.9679 | 0.8117 |
| 2006 | 0.9173 | 0.2531 | 1.3238 | 4.5726 | 4.6877 | -5.3877 | -2.3931 | 2.6055 | 0.8117 |
| 2007 | -0.8061 | 0.8123 | 0.7474 | 4.1880 | -0.2772 | -0.2070 | -1.8400 | 1.4130 | 0.8117 |
