# Supplementary material for: Inferring space from time: On the relationship between demography and environmental suitability in the desert plant O. rastrera
Source: PLoS One. 2018 Aug 9;13(8):e0201543. doi: 10.1371/journal.pone.0201543 (PMC6084933; doi:10.1371/journal.pone.0201543)
Supplement: S5 File — Three models were tested in every case: ID, a linear model relating the parameters with the climatic variables without any transformation; Log, as before, but log-transforming the bioclimatic variable; and Inv, in which the reciprocal (multiplicative inverse) of the bioclimatic variable was used. The model with the lowest AIC was selected and the function plotted over the whole range of climatic parameters observed in the study region. If the function showed an unusual behavior when extrapolated to the whole region (e.g., it resulted in biologically absurd parameters), we fitted two new models suggested by the form of the relationships observed: LogInv, in which the bioclimatic variables where log-transformed and their inverse calculated; and InvLogInv, which was as LogInv, but with an inverse link function. This procedure was only performed for the bioclimatic variable with the lowest AIC. We selected the function that was most similar to the one with the lowest AIC over the observed range of the bioclimatic variable but that produced biologically sensible estimates when extrapolated. In all the following tables we follow the next conventions: Numbers highlighted with yellow: Model used, Red numbers: Do not differ from the best model, Bold numbers in red: Best model but not necessarily the model used depending on AIC differences and following parsimony. For parameter θb, we kept the variable with greatest influence but used a different model than the one with lowest AIC because it resulted in biologically more meaningful results when extrapolating. For parameters αi, the model with the lowest AIC differ minimally from the null model (ΔAIC < 2) and for parsimony, we kept the null model. In the case of βm, the ΔAIC value between the null model and the best one was 2.03, but we still selected the null model. This was because the best model for βm implied the inclusion of the bioclimatic variable bio14 in the analyses thus increasing the computational time required to [file pone.0201543.s005.docx]

**S5. Data for the selection of the models that link demographic parameters to bioclimatic variables**. Three models were tested in every case: ID, a linear model relating the parameters with the climatic variables without any transformation; Log, as before, but log-transforming the bioclimatic variable; and Inv, in which the reciprocal (multiplicative inverse) of the bioclimatic variable was used. The model with the lowest AIC was then selected and the function plotted over the whole range of climatic parameters observed in the study region. If the function showed an unusual behavior when extrapolated to the whole region (e.g., it resulted in biologically absurd parameters) we fitted two new models suggested by the form of the relationships observed: LogInv, in which the bioclimatic variables where log-transformed and their inverse calculated; and InvLogInv, which was as LogInv, but with an inverse link function. This procedure was only performed for the bioclimatic variable with the lowest AIC. We selected the function that was most similar to the one with the lowest AIC over the observed range of the bioclimatic variable but that produced biologically sensible estimates when extrapolated.

In all the following tables we follow the next conventions: *Numbers highlighted with yellow*: Model used, *Red numbers*: Do not differ from the best model, *Bold numbers in red*: Best model but not necessarily the model used, depending on AIC differences and following parsimony. For parameter *θ*_b_ we kept the variable with greatest influence, but used a different model than the one with lowest AIC because it resulted in biologically more meaningful results when extrapolating. For parameters *α*_i_ the model with the lowest AIC differ minimally from the null model (ΔAIC < 2) and for parsimony we kept the null model. In the case of *β*m, the ΔAIC value between the null model and the best one was 2.03, but we still selected the null model. This was because the best model for *β*m implied the inclusion of the bioclimatic variable bio14 in the analyses, thus increasing the computational time required to produce the kernels for each pixel in the map by months.

**S5 Table A**

**S4 Table B**

**S5 Table C**

**S5 Table D**
